# P-Selectin Glycoprotein Ligand 1: A Potential HIV-1 Therapeutic Target

**DOI:** 10.3389/fimmu.2021.710121

**Published:** 2021-08-09

**Authors:** Silvere D. Zaongo, Yanqiu Liu, Vijay Harypursat, Fangzhou Song, Huan Xia, Ping Ma, Yaokai Chen

**Affiliations:** ^1^Division of Infectious Diseases, Chongqing Public Health Medical Center, Chongqing, China; ^2^Basic Medicine College, Chongqing Medical University, Chongqing, China; ^3^Department of Infectious Diseases, Tianjin Second People’s Hospital, Tianjin, China; ^4^School of Medicine, Nankai University, Tianjin, China

**Keywords:** P-selectin glycoprotein ligand 1, HIV, therapeutic target, infection, inflammation

## Abstract

Antiretroviral therapy (ART), which is a life-long therapeutic option, remains the only currently effective clinical method to treat HIV-1 infection. However, ART may be toxic to vital organs including the liver, brain, heart, and kidneys, and may result in systemic complications. In this context, to consider HIV-1 restriction factors from the innate immune system to explore novel HIV therapeutics is likely to be a promising investigative strategy. In light of this, P-selectin glycoprotein ligand 1 (PSGL-1) has recently become the object of close scrutiny as a recognized cell adhesion molecule, and has become a major focus of academic study, as researchers believe that PSGL-1 may represent a novel area of interest in the research inquiry into the field of immune checkpoint inhibition. In this article, we review PSGL-1’s structure and functions during infection and/or inflammation. We also outline a comprehensive review of its role and potential therapeutic utility during HIV-1 infection as published in contemporary academic literature.

## Introduction

Despite several decades of dedicated research, and large financial and human resource investments, no single effective treatment has been developed to eliminate HIV infection (1). To date, the best approach to treat HIV infection remains the administration of antiretroviral therapy (ART), which remains a life-long commitment to the diligent daily ingestion of multiple antiviral drugs (2). However, in the long term, ART can be toxic to vital organs such as the liver, brain, heart, and the kidney (3–10), resulting in systemic physical complications. As such, several alternative therapeutic strategies are being investigated by researchers. It is well recognized that HIV-1 infection triggers pathogen-recognition receptors (PRRs) in infected cells, leading to recognition of viral pathogen-associated molecular patterns. PRRs subsequently initiate a signaling cascade working at restricting the replication and spread of the virus, using innate intracellular antiviral defenses. Such an intrinsic response from the infected cell is, thereafter, disseminated *via* the action of cytokines and chemokines, which activate and attract innate immune cells to the site of infection. These effector cells can thereby assist in the control of viremia, and regulate immune responses to HIV-1 (11). Unfortunately, in spite of having a well-developed innate immune system composed of restriction factors capable of interrupting the viral replication cycle at particular points, HIV-1 does successfully infect CD4 T-cells and macrophages (11–15). Nevertheless, the restriction factors (which are host proteins) still warrant thorough investigation for their potential therapeutic application. These restriction factors possess a few characteristics in common (16), which can be summarized as: (i) they are usually induced by interferons (IFNs), (ii) they are usually developed from lentiviruses, mounting high selection pressure, leading to a rapid evolution of their coding genes, and resulting in significant production of their amino acid sequences (17), and (iii) in HIV-1 infection, the virus can restrict their actions in order to survive in host cells. Identification of such restriction factors from innate immunity that are involved in HIV-1 modulation and/or control represents a promising approach for the discovery of novel therapeutics to treat HIV infection.

Tripartite motif-containing protein 5-α (TRIM5-α) (18), APOBEC3 (19), tetherin (20), SAM domain and HD domain-containing protein 1 (SAMHD1) (21, 22), and serine incorporator 3 (SERINC3)/SERINC5 (23, 24) are examples of a few restriction factors identified thus far. Recently, P-selectin glycoprotein ligand 1 (PSGL-1) has been identified as another restriction factor, and is generating considerable interest. Indeed, Ying Liu and colleagues revealed, in a proteomic profiling study comparing infected and normal CD4 T-cells, that PSGL-1 is downregulated during HIV-1 infection (25). PSGL-1 sparked the interest of these scientists as it (i) showed a very high likelihood of positive selection to HIV-1, (ii) was specifically expressed in HIV-1 target cells such as lymphocytes and myeloid cells, (iii) was proven to be downregulated in the membrane fraction of the HIV-1-infected CEM-T4 cell line (26), and (iv) was proven to be associated with HIV-1 Gag at HIV-1 assembly sites (27). Following further investigation, they demonstrated that interferon-γ anti-HIV activity is mediated by PSGL-1, and is, on the other hand, antagonized by the HIV-1 viral particle unit (Vpu) (25). Of late, studies of this molecule have shown that PSGL-1 has a novel role in the area of immune checkpoint inhibition (28). PSGL-1 was, for a long time, studied as an adhesion molecule (29, 30), and is now progressively recognized as an important regulator of many facets of immune responses by myeloid cells and T-cells in settings of homeostasis and inflammation (31, 32). Furthermore, the role and potential therapeutic implications of PSGL-1 during HIV-1 infection has become an area of intense focus in academic research. A more thorough understanding of PSGL-1 and its potential functions and functional roles, as described in particular contexts, is warranted.

Herein, we review critical information related to PSGL-1, such as its structure and roles during infections and/or inflammation. We conclude this article with a review of the most recent knowledge related to the role of PSGL-1 during HIV-1 infection.

## Definition and Structure

P-selectin glycoprotein ligand 1 (PSGL-1) is coded by a human gene called Selectin P ligand, also known as SELPLG. The protein is also referred to CD162. PSGL-1 is a disulfide-bonded homodimeric type I transmembrane mucin-like glycoprotein, of 120 kilodaltons (KDa) in molecular weight (33–36) that is expressed on most peripheral T-cells and on some B-cells, neutrophils, monocytes, and platelets (37). Upon further post translational modifications (described below), PSGL-1 acquires the capacity to bind P-selectin, which is one of the selectin family of cell adhesion molecules, including E-selectin (endothelial) and L-selectin (leukocytes). The N-terminus of the extracellular domain is necessary to bind P- (with highest affinity), E-, and L-selectin (38–40) through varying levels of affinity (41–45). P-selectin is expressed on platelets and activated endothelial cells (46).

PSGL-1 requires sialyl Lewis x (sLex)-capped O-glycans to bind selectins. This glycosylation is dependent upon glycosyltransferases (including fucosyltransferase-VII, FucT-VII); sialyl 3-galactosyltansferases, ST3Gal-IV and ST3Gal-VI; and β1,4-galactosyltransferase, β1,4GalT-I) (47, 48) sequentially adding carbohydrate moieties to form sLex, which is the dominant sialylated fucosylated O-linked glycan. sLex thereafter mediates selectin binding, with specific enzyme requirements for each of the selectins (47, 49) (**Table 1**). The enzymes required for PSGL-1 and selectin binding are constitutively expressed by myeloid cells (61), T-cell progenitors (62), and hematopoietic stem cells (HSCs) (38, 63). Although PSGL-1 is highly expressed on resting T cells, selectin binding ability is only conferred during effector T-cell proliferation and differentiation (64, 65). Sulfation of tyrosine residues at the N-terminus of PSGL-1 is necessary for the binding of P- and L-selectin. PSGL-1’s cytoplasmic domain interacts with ezrin/radixin/moesin (ERM) proteins, attaching PSGL-1 to the actin cytoskeleton. PSGL-1 and ERM-family proteins are found in the uropods of polarized leukocytes. The activation of the Src-family kinases Fgr, Hck, and Lyn occurs when PSGL-1 is engaged by P-selectin or E-selectin (66, 67). Interestingly, PSGL-1 must be localized to leukocyte microvilli in order to bind its ligands optimally (68). Structurally, the cytoplasmic and trans-membrane domains of PSGL-1 are both strongly conserved (49), suggesting important functions. PSGL-1’s cytoplasmic domain, which consists of 72 and 69 amino acids for humans and mice, respectively (69), is critical for interactions with scaffolding and signaling molecules (70). Despite variations in PSGL-1’s extracellular domain between organisms, its overall structure and function tend to be regulated in a similar way (71). Regarding PSGL-1 as a therapeutic target, it is critical to understand the various functions that PSGL-1 plays depending on the specific infective and/or inflammatory context involved.

**Table 1:** Selectins expression and the enzymes required for PSGL-1 binding.

Type of ligand	Expressed on Cell/Tissue	References	Reported Glycosyl- and sulfotransferases involved in selectin-ligand biosynthesis
P-selectin	Choroid plexus	(50)	Fucosyltransferase-VII (FucT-VII), Fucosyltransferase-IV (FucT-IV), Core 2 β1,6-glucosaminyltransferase (C2GlcNAcT-I), Sialyl 3-galactosyltransferase (ST3Gal), β1,4-galactosyltransferase (β1,4GalT-I), Tyrosine protein sulfotransferases 1,2 (TPST1,2)
	Lung endothelium	(51)	
	Platelets	(52)	
	Platelet-derived microparticles	(53)	
	Peritoneal macrophages	(54)	
	Inflamed endothelium	(55, 56)	
E-selectin	Skin endothelium	(57)	Fucosyltransferase-VII (FucT-VII), Fucosyltransferase-IV (FucT-IV), Sialyl 3-galactosyltransferase (ST3Gal)
	Inflamed endothelium	(55)	
L-selectin	Myeloid cells	(55)	Fucosyltransferase-VII (FucT-VII), Fucosyltransferase-IV (FucT-IV), Core 2 β1,6-glucosaminyltransferase (C2GlcNAcT-I), Sialyl 3-galactosyltransferase (ST3Gal), Tyrosine protein sulfotransferases 1,2 (TPST1,2)
	Naive T-cells	(55)	
	Effector T-cells	(58)	
	Effector memory T-cells	(58)	
	Central memory T-cells	(58)	
	Monocytes	(59)	
	Neutrophils	(60)	

## Recognized PSGL-1 Functions During Infections and/or Inflammation

**Figure 1:**
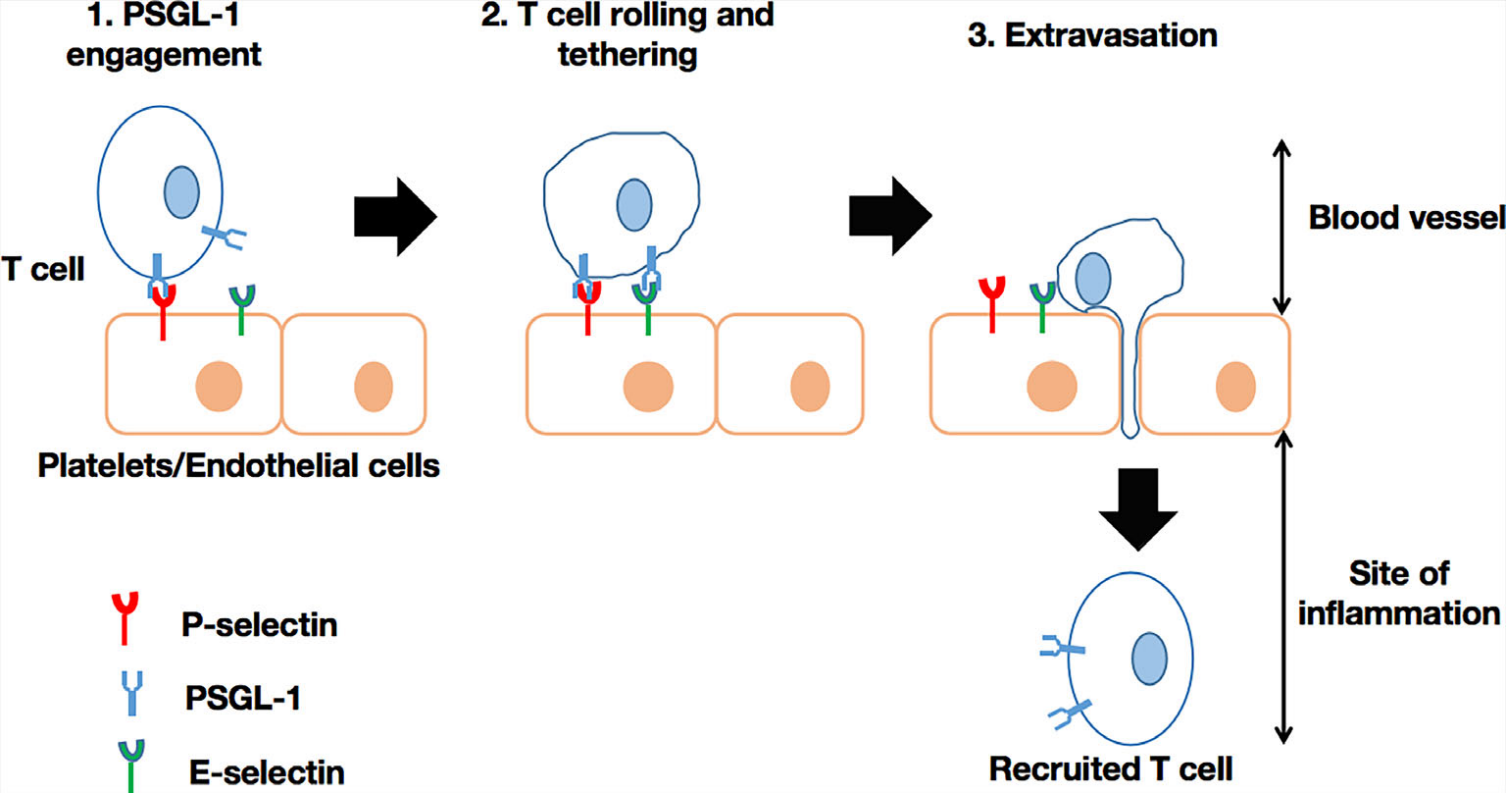
T-cell recruitment *via* PSGL-1/selectin binding. In the blood vessel, the circulating T-cell is engaged by selectin on platelets (P-selectin) or endothelial cells (P-selectin or E-selectin). Subsequently, the T-cell is able to initiate rolling and the extravasation cascade, leading to its recruitment into the site of inflammation, where it can intervene as an effector cell. This process can also take place in the recruitment of other leukocytes.

Past *in vitro* experiments have revealed that PSGL-1 regulates neutrophil rolling and tethering on activated endothelium through P-selectin binding. Later studies using *Selplg* ^-/-^ murine cells (72), confirmed the role of PSGL-1 in modulating the motility of macrophages/monocytes, plasma B-cells, dendritic cells, and T-cells through selectin engagement (47, 49). Thus, PSGL-1 is able to (i) facilitate neutrophil migration into inflamed peritoneum (73), (ii) recruit CD8+ T-cells into the inflamed colon (74), or (iii) recruit CD4+ T-cells into responding lymph nodes (75), intestinal lamina propria (76), and the inflamed retina (77). In short, PSGL-1 plays a critical role in the recruitment of immune cells into sites of inflammation (**Figure 1**). However, PSGL-1 mutation with the absence of an intracellular tail in cells may result, for instance, in significantly reduced rolling efficiency (70). Moreover, PSGL-1 is involved in neutrophil and monocyte localization. For instance, PSGL-1 mediates the adhesion of these cells to P-selectin, which is expressed on platelets that become attached to inflamed endothelium. Such interactions can enable targeted extravasation into tissues by eliciting chemokine secretion by platelets, as well as inflammatory mediator production by neutrophils (78). Also, neutrophils, *via* PSGL-1 expressed on their cell membrane, can bind to platelets *via* P-selectin to achieve extravasation (79). PSGL-1 on other immune cells can interact with P-selectin expressed on endothelial cells to promote extravasation, especially if the inflammation results from the activation by thrombin or histamine (46). PSGL-1 has been implicated in the establishment of cellular complexes that function in pathogen clearance has been suggested, as platelets also utilize PSGL-1 to adhere to vasculature by selectin binding (80). Illustration of this is the host response to *Salmonella typhimurium* where platelet PSGL-1/P-selectin interaction leads to neutrophil recruitment (81). With regards to the role of PSGL-1 in immune responses, microbes have the capacity to develop mechanisms to neutralize the effects of PSGL-1 by targeting selectin-binding by PSGL-1. Of note, it has been demonstrated that the sialic acid-binding toxin, staphylococcal superantigen-like 5 (SSL5) (secreted by *Staphylococcus aureus*), has the ability to bind to sLex expressed on PSGL-1 on neutrophils. Consequently, neutrophils can neither be activated nor recruited *via* PSGL-1/P-selectin binding (82, 83). Due to several endogenous mechanisms, PSGL-1’s role in immune cell recruitment can either be enhanced or inhibited. For example, interactions between PSGL-1 and (i) Sialic acid-binding immunoglobulin-type lectin 5 (Siglec 5) (84) or ADAM8 (proteolytic enzyme) (85) on one hand, and (ii) ADAM28 (86) on the other, were shown to inhibit/block leukocyte rolling on P- and E- selectin, but enhanced binding to P-selectin, respectively.

PSGL-1, in addition to its role in cell migration, was demonstrated to be an inhibitor of human HSCs proliferation subsequent to ligation by P-selectin or anti-PSGL-1 antibody *in vitro* (87). On dendritic cells (DCs), PSGL-1 engagement by P-selectin or anti-PSGL-1 antibody was associated with immune inhibition, and an enhanced capacity to induce regulatory T-cells *in vitro*. Such engagement stimulates the production of c-Fos, indoleamine 2,3 dioxygenase (IDO), interleukin 10 (IL-10), and transforming growth factor (TGF)-β, all recognized for their roles as regulator of cell function (88). PSGL-1 ligation can also stimulate cytokine release by macrophages, DCs and T-cells leading to beneficial responses to pathogens (88–92). Further functions have been attributed to PSGL-1 in microbial infections. Notably, it assists in the control of *Streptococcus pneumoniae* by supporting neutrophil phagocytosis through binding to the capsular polysaccharide and cell wall autolysin, LytA (93). On the other hand, Enterovirus 71 has been shown to utilize PSGL-1 selectin-binding domain as a primary receptor to infect leukocytes, which aids viral replication (94). Furthermore, PSGL-1 possesses broad-spectrum antiviral activity (against e.g., HIV-1, murine leukemia virus, and influenza A virus) (95), by blocking viral infections through steric hindrance of particle attachment to target cells. A recent study shows that the expression of PSGL-1 in virus-producing cells impairs the incorporation of the SARS-CoV and SARS-CoV-2 spike (S) glycoproteins into pseudovirions, and blocks virus attachment and subsequent infection of target cells (96). Thus, *via* PSGL-1, bacteria or viruses may promote or inhibit the immune response. **Table 2** summarizes the roles of PSGL-1 as reported during some bacterial and viral infections.

**Table 2:** Summary of the roles of PSGL-1 in bacterial and viral infections.

Type of infection	Example of pathogen	Reported process	Reference
Bacteria	*Salmonella typhimurium*	PSGL-1/P-selectin interactions lead to neutrophil recruitment and host defenses	(81)
	*Staphylococcus aureus*	Staphylococcal superantigen-like 5 can bind sLex expressed on PSGL-1 by neutrophils. Therefore, neutrophils cannot be activated or recruited *via* PSGL-1/P-selectin binding	(82, 83)
	*Streptococcus pneumoniae*	PSGL-1 is involved in neutrophil phagocytosis through binding to the capsular polysaccharide and cell wall autolysin, LytA	(93)
Viruses	SARS-CoV and SARS-CoV-2	PSGL-1 impairs the incorporation of the viral spike (S) glycoproteins into pseudovirions. Thus, it blocks virus attachment and subsequent infection of target cells	(96)
	Murine leukemia virus	PSGL-1 inactivates murine leukemia virus infectivity	(95)
	Influenza A virus	PSGL-1 disrupts the infectivity of this nonretroviral enveloped virus	(95)
	HIV	In the presence of PSGL-1, the produced HIV particles harbor a defective membrane (without gp120 and gp41). They are therefore ineffective at binding to target cells	(25, 95)
		PSGL-1 cytoplasmic domain (T393) binds F-actin and, therefore, restricts cellular actin dynamics. Without F-actin to recruit, HIV cannot complete the reverse transcription process.	(97)

Finally, a recent publication extensively reviewed the role of PSGL-1 in the development of neoplastic disease. The authors reported that blocking the CD4^+^ T-cell’s PSGL-1 pathway could possibly be utilized as a novel cancer therapeutic approach to treat tumors (98). Indeed, PSGL-1 activation in the tumor microenvironment can promote CD4^+^ T-cell exhaustion pathways, which promotes tumor development. Therefore, ligands including chemokines CCL19 and CCL21 (62, 99, 100), Siglec-5 (84, 101), versican, and V-domain immunoglobulin suppressor of T cell activation (VISTA) (102) could possibly bind PSGL-1 on T-cells, and could contribute to inhibitory signaling pathways that dampen T-cell receptor (TCR) signals to induce T-cell exhaustion in tumors. However, there is currently no evidence that blocking PSGL-1 on CD4 T-cells alone would be sufficient to promote tumor growth control without also relieving exhaustion of CD8 T-cells. Further investigation will be necessary to understand the biology of PSGL-1 on T cells as well as other immune cells in tumors.

## PSGL-1 and HIV-1 Infection

Within these last two years, studies on PSGL-1 and its putative roles during HIV-1 infection have increased dramatically. Investigations by HIV researchers into PSGL-1 have been stimulated after a study by Liu et.al., was published (25), showing that PSGL-1, which can be induced by interferon-γ in activated CD4+ T-cells, inhibits HIV-1 reverse transcription, and effectively blocks viral infectivity. The same research team then demonstrated that HIV-1 Vpu downregulates PSGL-1 expression in infected CD4+ T-cells after binding to it and inducing its ubiquitination and degradation through the protein ubiquitin ligase SCF^β-TrCP2^. To further illustrate the downregulation of PSGL-1 during HIV-1 infection, it is worth considering earlier results of Liang and colleagues (103). They compared PSGL-1 expression on monocytes from healthy individuals (n=26) and treatment-naive participants with primary HIV-1 infection (PHI, n=38) or chronic HIV-1 infection (CHI, n=20). They found that PSGL-1 expression was significantly decreased in the PHI group (P<0.001). PSGL-1 expression on monocytes was partially restored in the CHI group, but did not return to the levels of healthy controls. These results suggest a negative association between PSGL-1 expression and HIV-1 viral load profiles. Indeed, the viral load profile (log_10_ copies/mL) was almost 4 times higher in the PHI cohort (4.21 ± 0.95) compared to the CHI cohort (1.30 ± 1.68). However, broader, and more in-depth investigations are required to be certain of this possible correlation and its likely determinants, such as age, infection duration, sex, and HIV-1 clades. In contrast, Connor et al., have found that compared with healthy patients, monocytes derived from HIV-infected individuals exhibit greater levels of PSGL-1. In their study, all HIV-infected participants (HIV positive for a longer period of time than in Liang et al.’s study) were under ART (longer than in Liang et al.’s study) suggesting the positive contribution of treatment to PSGL-1 complete restoration and expression (average mean ~1 for HIV- *versus* ~1.5 for HIV+, p=0.0204). They also observed that c-Myc regulates PSGL-1 expression in monocytes during HIV-1 infection, and that glutamate and sCD40L can promote PSGL-1 expression (104). During their study, Liang et.al., did not analyze the effects of HIV-1 or of its associated particles (Vpu, Vpr and Nef) to explain PSGL-1 disparities observed in HIV-1 positive participants. Five years later, Liu et.al., demonstrated for the first time that overexpression of Vpu but not Nef, Vif, Vpr, nor p55 Gag, significantly decreased PSGL-1 levels in 293T cells, Jurkat cells, and CD4+ T-cells from two donors (25). Although their findings were novel, they looked at intracellular PSGL-1 levels through mRNA profiling only. Just months after the findings of Liu et.al., Fu et.al., provided further details. After confirming that only Vpu negatively regulates intracellular PSGL-1, they also discovered that Vpu and Nef negatively regulates extracellular PSGL-1 levels (95). To date, it is well known that HIV-1 can efficiently suppress PSGL-1’s ability to block viral infectivity by repressing its expression. Fu’s team offered additional information on this process, which was first stated by Liu *et al.* They confirmed that PSGL-1 in virus-producer cells was not blocking the release of novel viral particles, but did inactivate virion infectivity. In other words, PSGL-1 in the virus-producer cells reduces levels of HIV-1 Env on virions. Consequently, PSGL-1 can inhibit virion attachment to cells in an envelope glycoprotein-independent manner. In fact, without HIV-1 Env structures required for cell attachment, such as gp120 and gp41 (105), novel particles are rendered ineffective at binding to target cells.

**Figure 2:**
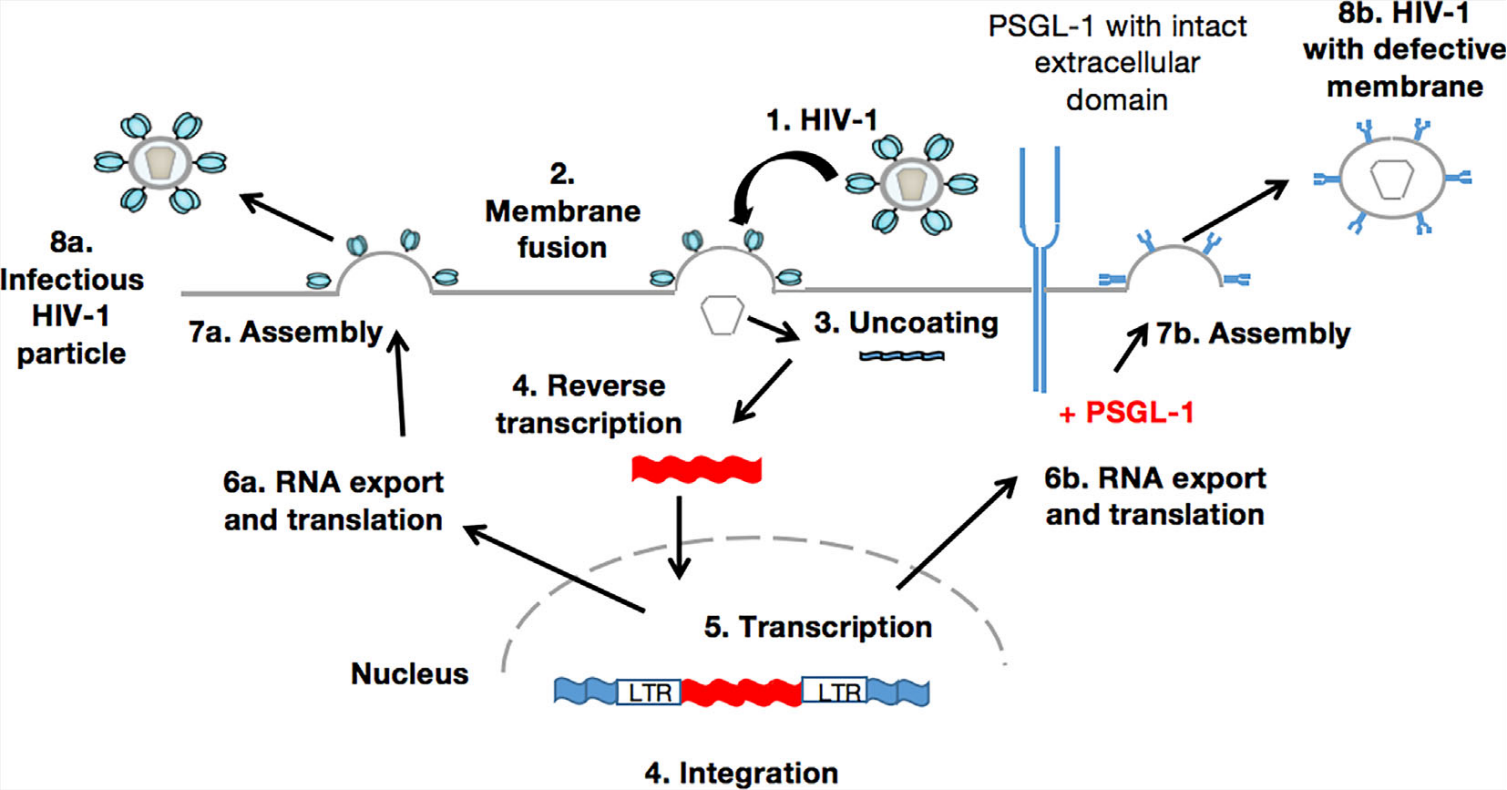
PSGL-1 mediates the production of membrane defective HIV-1 particles. The infected target cell (1-5) produces viral RNAs and proteins that are normally assembled (6a-8a). These new viral particles can infect new cells in which the HIV-1 replication cycle would therefore take place. Conversely, in the presence of PSGL-1 (6b-8b), novel viral particles possess a defective membrane within which PSGL-1 is incorporated. Such a phenotype leads to the production of membrane defective virions that, in the absence of crucial elements such as gp120 and gp41, are unable to attach to or infect new target cells.

**Figure 3:**
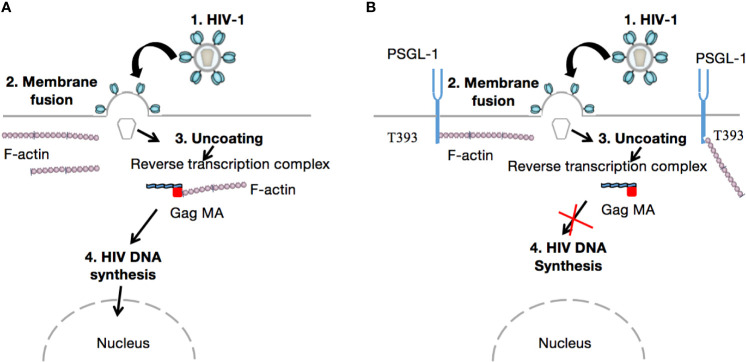
Inhibition of HIV-1 reverse transcription. After attachment of the viral particle to the target cell and incorporation of the inner core into the cell cytoplasm, viral RNA is released (Uncoating). Thereafter, reverse transcription takes place in the cytoskeletal compartment. The viral reverse transcription complex interacts with F-actin *via* its gag MA domain to achieve cDNA synthesis **(A)**. However, in the presence of PSGL-1, which binds F-actin *via* its T393 conserved cytoplasmic domain, actin depolymerization or interaction with gag MA within the viral reverse transcription complex cannot occur **(B)**. Consequently, HIV-1 cannot recruit intact actin microfilaments to complete reverse transcription; as reported by Bukrinskaya A et al., in 1998 (107).

When the HIV virion has found target cells (CD4 T-cells), the virus surface glycoprotein (gp120) binds to a cell receptor or co-receptors such as chemokine CC receptor 5 (CCR5) or chemokine CXC receptor 4 (CXCR4), also responsible for the HIV entry into lymphocytes and macrophages. The binding of gp120 results in the exposure of gp41, bringing the HIV virion much closer to the target cell. Subsequently, there is fusion of the viral envelope with the host cell membrane, which is essential for the entry to the inner matrix core of the virus (105). Within this inner core are two strands of viral RNA held together by two small proteins (P6 and P7), three of the viral enzymes (integrase, protease, and reverse transcriptase), and accessory proteins such as Nef, Vpr, and Vif. The viral RNA then undergoes conversion into viral DNA through a process mediated by viral reverse transcriptase before its integration into the host cell genome. In the absence of gp120 and gp41, the aforementioned process does not take place. Importantly, PSGL-1 is incorporated into progeny virions which explains the loss of infectivity, as HIV-1 Env is disorganized by this presence (**Figure 2**). It is worth noting that the PSGL-1 extracellular domain is fundamental to this process. Fu and colleagues reported that the deletion of much of the intracellular C-terminal domain had no impact on PSGL-1’s ability to restrict HIV-1. On the contrary, the truncation of the extracellular N-terminal domain effectively eliminated the restrictive phenotype. This observation was revealed one month earlier *via* an e-publication by a Japanese team of researchers (106), who also demonstrated that co-clustering of Gag with PSGL-1 [previously shown to be dependent on the MA domain, especially the HBR (27)] and subsequent release of progeny virions, promote extracellular PSGL-1 down-regulation in infected cells. Very recently (August 2020), Liu et.al (97)., revealed a series of findings showing that PSGL-1 binds to cellular actin filaments (F-actin) to restrict actin dynamics, which consequently inhibit HIV-1 DNA synthesis and HIV-1 reverse transcription (**Figure 3**). Of note, PSGL-1’s inhibition of HIV-1 reverse transcription is dependent on the cytoplasmic domain, which by itself is sufficient for the inhibition. Importantly, IFN-γ mediates PSGL-1 anti-HIV-1 activity (25), but also increases F-actin intensity/distribution. However, the intensity of F-actin was largely eliminated when PSGL-1 was knocked down by siRNA electroporation. F-actin intensity is inversely related to HIV-1 infection, implying that PSGL-1’s anti-HIV-1 activity is linked to its regulation of F-actin intensity. PSGL-1 cytoplasmic domain binds F-actin and inhibits its depolymerization. Indeed, a highly conserved threonine (T393) was identified as a key residue for the binding of PSGL-1 to actin. Subsequently, PSGL-1’s inhibition of reverse transcription and ability to increase F-actin intensity were greatly reduced by a T393 mutation (97). This represents PSGL-1’s early inhibition role in HIV-1 infection, and is a plausible explanation for this process as it is known that an actin cytoskeleton is required for HIV reverse transcription (107).

With regards to the aforementioned roles that PSGL-1 plays during HIV infection, it is important to inform on its potential applications. Firstly, PSGL-1 can inhibit HIV-1 DNA synthesis and virion infectivity. Compared with HIV DNA synthesis, infectivity inhibition appears to be more potent. Indeed, significant inhibition of virion infectivity can be achieved with a low level of PSGL-1 overexpression (25). Liu and colleagues reported that Vpu has the ability to repress PSGL-1 expression; however we believe that further studies aiming at understanding the interactions between Vpu and PSGL-1 may help develop new anti-HIV drugs. In 2009, Ohba et al. demonstrated that follicular dendritic cells (FDCs) can efficiently activate HIV-1 replication in latently infected monocytes/macrophages. They found that interaction of PSGL-1 with P-selectin is involved in this process, and they further suggested a linkage between PSGL-1 signaling and HIV-1 replication through the activation of nuclear factor k-light chain-enhancer of activated B cells (NF-kB). Indeed, when PSGL-1 on infected monocytes/macrophages interacts with P-selectin on FDCs, a juxtacrine signaling mechanism [with spleen tyrosine kinase (Syk) acting as a downstream effector of PSGL-1 during HIV-1 replication] is activated, and therefore facilitates HIV-1 replication (108). However, this observation is only applicable to monocytes/macrophages as Ohba et al., (in their unpublished data) have found that the PSGL-1/Syk pathway in CD4 T-cells was not prominent. Although this finding seems to discount PSGL-1’s repressive role (reported in contemporary literature, as detailed above) during HIV infection, one may speculate on the benefits of its ability to trigger latently infected cells. We believe that PSGL-1 could be an alternative to latency reversal agents (LRAs) in the “shock and kill” strategy. Such an approach could provide additional tools to finding a cure to HIV infection, especially since it is known that reservoir cells are one of the major impediments to HIV treatment. Secondly, PSGL-1 is an HIV restriction factor, and as such, its expression is triggered by interferon-γ, an immunomodulatory cytokine whose role in innate immunity in HIV infection has only begun to be elucidated (109, 110). Additionally, IL-12 (using Th1 cells from mice) (98), soluble CD40 ligand (sCD40L, using monocytes from HIV negative donor) (104), and glutamate (using monocytes from HIV negative donor) (104) have been proven to be capable of inducing PSGL-1 expression *ex vivo*. Thus, interferon-γ or IL-12 administration in combination with strategies such as ART, immunotherapy, and gene therapy may provide substantial improvement in the immune responses of HIV-infected individuals. By neutralizing HIV’s repressive role on PSGL-1 expression, immune cells can more readily be recruited to participate in controlling and ameliorating inflammation and/or infection. Importantly, it is now known that during HIV infection there is a fundamental imbalance in the gut microbiome, with a depletion of key immune cells involved in regulating gut microorganisms. CD4+ T-lymphocytes in the gut-associated lymphoid tissues (GALT) (111), Paneth cells (112), macrophages (113), epithelial cells (114), and Th17 cells (115) are all aberrantly represented during HIV infection. Promoting PSGL-1 expression could help in recruiting new cells of the preceding types into the gut associated tissues and re-establishing immune homeostasis, which depends on the overall integrity of the gut microbiome (116), especially in immunological nonresponders. And thirdly, c-Myc regulates PSGL-1 expression in monocytes during HIV-1 infection (104) and this is important, particularly for the treatment of complications associated with HIV, including cardiovascular diseases (CVDs) and neurocognitive disease. It has been reported that sCD40L is implicated in the development of disorders such as inflammatory bowel disease and atherosclerosis (117, 118). Glutamate dysregulation has been demonstrated in a variety of neurocognitive disorders (119). Since sCD40L and glutamate have been proven to be effective at inducing PSGL-1 expression on monocytes (104), an approach targeting PSGL-1 using these factors during HIV infection could be a viable alternative approach to the treatment of CVDs and neurocognitive disease.

## Conclusion

Knowledge gaps remain regarding PSGL-1 expression (both intra- and extracellular) and its functions during HIV-1 infection. For example, the impact of HIV-1 clades on PSGL-1 activities requires clarification. Considering that F-actin intensity in infected cells is inversely related to HIV-1 infection, as reported (97), we may therefore suppose that PSGL-1 activity depends upon HIV-1 strain. In other words, PSGL-1 repression is stronger as the strain becomes more virulent. Yet a question remains viz., what are the most repressive viral strains/clades? This is an area that warrants further study. Further questions, such as whether PSGL-1 is an immunological determinant in elite controllers (120–122), long-term non-progressors (123, 124), and immunological non-responders (125, 126), require robust and accurate answers. Finally, since PSGL-1 is expressed by a variety of myeloid cells and T-cells (CD4 and CD8), it is crucial to investigate any potential associations between T-cells and PSGL-1 during HIV infection. Perhaps, a simple index such as the humble CD4+/CD8+ ratio, which is essential for tracking HIV-1 progression (127), could possibly be recruited to track PSGL-1 expression. PSGL-1 is certainly a potential novel therapeutic target for HIV-1, but is also a potential therapeutic target for the treatment of other viral infections as well, when considering its broad-spectrum activities. Further targeted investigation is therefore required to achieve this goal.

Although several studies suggest the benefits of PSGL-1 expression on immune cells during infection/inflammation, some studies present a dissenting picture. For instance, Tinoco et al., have reported that PSGL-1 acts as a negative regulator of T-cell function, which may be exploited by a chronic viral infection to inhibit effector T-cells. They observed that PSGL-1 ligation on exhausted CD8 T-cells (1) inhibits TCR expression, and IL-2 signaling, and (2) upregulates the programmed cell death protein 1 (PD-1) (128). The consequence is their diminished survival with TCR stimulation. Additionally, promoting PSGL-1 expression by DCs, for instance, could limit their immunostimulatory capacity. Indeed, it is known that cDC1 dendritic cells are required to effectively activate both CD4+ and CD8+ T-cells in cellular responses to tumors (129). However, PSGL-1’s role in T-cell activation when expressed on DCs is unknown. Nevertheless, studies have shown that P-selectin engagement with DCs can induce a tolerogenic phenotype that can suppress T-cells (88). These data suggest that PSGL-1, as a potential therapeutic option for HIV, should be explored with caution.

## Author Contributions

SZ wrote the manuscript and conceived the figures. VH and YC revised, co-wrote, and provided significant inputs. All authors contributed to the article and approved the submitted version.

## Funding

This work was funded by the Medical Research Project of Chongqing Municipal Science and Technology Bureau and Health Commission (grant number 2020GDRC004) and the Key Medical Research Project of Chongqing Municipal Science and Technology Bureau and Health Commission (grant number 2019ZDXM012).

## Conflict of Interest

The authors declare that the research was conducted in the absence of any commercial or financial relationships that could be construed as a potential conflict of interest.

## Publisher’s Note

All claims expressed in this article are solely those of the authors and do not necessarily represent those of their affiliated organizations, or those of the publisher, the editors and the reviewers. Any product that may be evaluated in this article, or claim that may be made by its manufacturer, is not guaranteed or endorsed by the publisher.

## Abbreviations

ADAM8, A Disintegrin and metalloproteinase domain-containing protein 8; ADAM28, A Disintegrin and metalloproteinase domain-containing protein 28; ART, Antiretroviral therapy; β1, 4GalT-I, β1, 4-galactosyltransferase; C2GlcNAcT-I, Core 2 β1, 6-glucosaminyltransferase; CCL19, Chemokine (C-C motif) ligand 19; CCL21, Chemokine (C-C motif) ligand 21; CD4, Cluster of differentiation 4; CD8, Cluster of differentiation 8; DCs, Dendritic cells; ERM, Ezrin/radixin/moesin; F-actin, Actin filaments; FucT-IV, Fucosyltransferase-IV; FucT-VII, Fucosyltransferase-VII; Gag, Group-specific antigen; HBR, Highly basic region; HIV-1, Human immunodeficiency virus type 1; HSCs, Hematopoietic stem cells; IDO, Indoleamine 2, 3 dioxygenase; IL-10, Interleukin 10; INFs, Interferons; KDa, Kilodalton; MA, Matrix protein; Nef, Negative factor; PRRs, Pathogen-recognition receptors; PSGL-1, P-selectin glycoprotein ligand 1; SARS-CoV, Severe Acute respiratory syndrome coronavirus; SARS-CoV-2, Severe Acute respiratory syndrome coronavirus 2; Siglec 5, Sialic acid-binding immunoglobulin-type lectin 5; sLex, Sialyl Lewis x; SSL5, Staphylococcal superantigen-like 5; ST3Gal, Sialyl 3-galactosyltransferase; Syk, Spleen tyrosine kinase; TCR, T-cell receptor; TGF, Transforms growth factor; T393, Threonine 393; TPST1, 2, Tyrosine protein sulfotransferases 1, 2; Vif, Viral infectivity factor; VISTA, V-domain immunoglobulin suppressor of T cell activation; Vpu, Viral particle unit; Vpr, Viral protein r.
